# Knowledge and Awareness of Noncommunicable Diseases and Influential Lifestyle Factors Among Physiotherapy Students: A Cross-Sectional Study

**DOI:** 10.7759/cureus.105010

**Published:** 2026-03-10

**Authors:** Manoj Kumar, Srisaisantoshini Sankaranarayanan, Shwetali Malunjkar, Shekhar Jambure

**Affiliations:** 1 Cardiopulmonary Physiotherapy, Mohamed Sathak A.J College of Physiotherapy, Chennai, IND; 2 Cardiopulmonary Physiotherapy, Dr. N. Y. Tasgaonkar College of Physiotherapy, Karjat, IND

**Keywords:** awareness on ncds, coronary artery disease (cad), diabetes, hypertension, modifiable risk factors, noncommunicable diseases (ncds), physical activity behavior

## Abstract

Introduction

Noncommunicable diseases (NCDs) represent a leading global health challenge, with modifiable lifestyle factors contributing substantially to their occurrence. Physiotherapists play a major role in supporting prevention and health promotion within the healthcare system, in addition to rehabilitation. Assessing physiotherapy students’ awareness, knowledge, and associated lifestyle factors is essential to prepare them for evolving public health roles.

Methods

This cross-sectional study was conducted among 286 students from multiple colleges. Data were collected using a structured questionnaire adapted from previous literature and shared through Google Forms (Google LLC, Mountain View, CA, USA). Descriptive statistics were used to summarize awareness and knowledge levels. The chi-square test evaluated the association between knowledge levels and year of study, with significance set at p < 0.05.

Results

Among 286 participants, 201 students (70.28%) were familiar with NCDs, while only 46 students (16.08%) had attended workshops. Most students relied on social media (n = 146, 51.05%) and faculty (n = 110, 38.46%) for information. About 86 students (30.07%) were aware of the WHO Global Action Plan. Overall, 230 students (80.4%) had moderate knowledge, 52 students (18.2%) had poor knowledge, and four students (1.4%) had good knowledge. Awareness of modifiable risk factors was high, with 265 students (92.66%) identifying the role of physical activity in prevention. Knowledge levels significantly improved with each academic year.

Conclusions

Physiotherapy students exhibited moderate overall knowledge of NCDs and associated lifestyle risk factors, with higher academic years showing improved awareness. Despite a strong understanding of lifestyle prevention, formal exposure to NCD-related training, workshops, and global initiatives remains limited. Integration of structured learning on NCD prevention and health promotion into the physiotherapy curriculum is recommended to strengthen students’ preparedness for public health responsibilities.

## Introduction

Noncommunicable diseases (NCDs) are the leading cause of death and disability worldwide, driving a global health focus on their prevention and management. NCDs account for over 70% of global deaths, with the highest mortality rates in low- and middle-income countries. Major contributors to NCD-related deaths include cardiovascular diseases, diabetes, cancers, and chronic respiratory conditions [1]. Despite ongoing efforts, most countries are not on track to achieve the WHO target of reducing premature deaths from NCDs by 2030 [2].

A large proportion of NCDs is attributable to modifiable lifestyle risk factors such as physical inactivity, poor diet, tobacco use, and chronic stress. Physical inactivity alone significantly increases the risk of cardiovascular diseases, type 2 diabetes mellitus, and certain cancers [3]. Poor dietary habits, characterized by low intakes of fiber and protein and high intakes of processed foods, are associated with an increased risk of NCDs [4]. Tobacco use remains a leading cause of NCD risk, significantly contributing to cardiovascular diseases and cancers [5]. Chronic stress also plays a critical role by contributing to physiological imbalances, immune dysfunction, and unhealthy behaviors such as smoking and poor diet, thereby compounding overall risk [6]. These risk factors often cluster together, significantly increasing the likelihood of developing NCDs. Therefore, preventive strategies targeting these lifestyle factors are essential and can be implemented at both individual and community levels to reduce the burden of NCDs.

Lifestyle modifications, including increased physical activity, adoption of a healthy diet, smoking cessation, and stress management, have proven effective in the prevention and control of NCDs [7]. Physiotherapists play a vital role in NCD prevention through structured exercise prescription, patient education, and facilitation of behavior change [8-10]. Evidence indicates that healthcare professionals with stronger knowledge of lifestyle-related risk factors are more effective in promoting adherence to healthy behaviors and supporting lifestyle modification [11].

Although physiotherapists are not primary providers of medical screening within the Indian public health system, their scope of practice in secondary and tertiary prevention includes functional assessment, identification of modifiable lifestyle-related risk indicators such as physical inactivity, obesity, and reduced functional capacity, as well as reinforcement of self-management strategies in clinical, community, and rehabilitative settings for conditions including cardiovascular disease, diabetes, and obesity [12,13]. These roles position physiotherapists as key contributors to risk mitigation rather than primary diagnosis.

Given this professional scope, competencies related to NCDs and associated lifestyle risk factors represent a must-know domain for secondary and tertiary prevention and a need-to-know domain for primary prevention within physiotherapy education. A strong foundation in NCD-related knowledge is therefore essential for physiotherapy students to effectively promote health, prevent disease progression, and educate patients on behavior modification [14]. However, existing physiotherapy curricula do not explicitly emphasize NCD prevention or lifestyle risk management, and there is limited published evidence describing the current level of awareness and knowledge of NCDs among physiotherapy students [15]. This study aims to assess awareness of NCDs and associated lifestyle factors among physiotherapy students, with the objective of identifying knowledge gaps and generating evidence to inform future educational and professional development strategies.

## Materials and methods

Study design and sample size

An exploratory, cross-sectional study was conducted to assess knowledge and awareness of NCDs and influencing lifestyle factors among physiotherapy students. The sample size was calculated using OpenEpi version 3 (sample size for frequency in a population) with an estimated population of 500 physiotherapy students across the participating colleges. The calculation was based on an expected prevalence of 50%, absolute precision of 5%, and a design effect of 1. The tool yielded a minimum sample requirement of 218 (95% CI) and 286 (99% CI). This calculation was intended for prevalence estimation and not for hypothesis testing of associations.

Participants

A total of 286 participants were selected using convenience sampling from accessible physiotherapy colleges in rural and urban regions. Students enrolled in an Indian university’s Bachelor of Physiotherapy (BPT) program from the first year (after six months of enrollment) to the internship period were included. Students enrolled in universities outside India, postgraduate Master of Physiotherapy students, and first-year students enrolled for fewer than six months were excluded. Participation was voluntary, and informed consent was obtained prior to data collection. A convenience sampling approach was adopted due to classroom-based access to eligible students across participating colleges during the study period.

Study instrument

Data were collected using a structured, self-administered questionnaire adapted from a previously published study conducted in Fiji [16]. The original questionnaire had undergone formal validity and reliability testing. For the present study, minor contextual adaptations were made to enhance geographic relevance. The demographic section was streamlined to include the course and year of study. Epidemiological data referring to NCD prevalence in Fiji were replaced with corresponding Indian prevalence data based on established epidemiological evidence from international and national reports, including the WHO and Ministry of Health and Family Welfare, Government of India [17,18]. Selected behavioral practice items were removed, as this study focused exclusively on knowledge and awareness. These modifications did not alter the underlying conceptual domains of the instrument. Content relevance and contextual clarity were reviewed by three senior physiotherapists with expertise in community health to ensure suitability for the Indian setting. The adapted questionnaire assessed: awareness of NCDs; knowledge of NCDs and their risk factors; lifestyle-related risk factors and prevention; and practical knowledge and readiness for action relevant to physiotherapy practice.

In this study, awareness and knowledge were treated as distinct constructs. Awareness was defined as prior exposure to or recognition of NCD-related concepts, whereas knowledge was defined as factual understanding of disease burden, risk factors, and prevention strategies, requiring correct responses [19]. Awareness items were analyzed descriptively and were not included in the composite score to avoid ceiling effects and misclassification. A composite knowledge score was computed from the knowledge-based items in the questionnaire to determine quantitative knowledge levels. Each correct response received a score of “1,” whereas each incorrect response received a score of “0.” Participants were divided into three knowledge groups based on their overall scores: poor knowledge (<40%), moderate knowledge (40-75%), and good knowledge (>75%) [20].

Data collection

The questionnaire was administered electronically using Google Forms (Google LLC, Mountain View, CA, USA) and distributed through official WhatsApp groups (Meta Platforms, Inc., Menlo Park, CA, USA) to eligible students. Because dissemination occurred through professional intermediaries, the exact number of students who received the survey invitation could not be determined; therefore, the response rate could not be calculated. Participation was voluntary, and electronic informed consent was obtained. To prevent duplicate responses, the form was restricted to one submission per Google account. Participants completed the questionnaire during class hours under faculty supervision and were instructed to complete it independently without referring to external sources. While absolute verification of independent completion was not feasible in online self-administered surveys, supervised in-class administration minimized the risk of external consultation and enhanced response authenticity. No identifiable information was collected, and responses were anonymized. Data collection was conducted over four weeks (July 1, 2025 to July 31, 2025), during which the survey link was active.

Statistical analysis

Statistical analysis was performed using IBM SPSS Statistics for Windows, Version 26.0 (Released 2018; IBM Corp., Armonk, NY, USA). Descriptive statistics were used to summarize awareness responses and knowledge levels. Associations between knowledge levels and year of study were examined using Fisher’s exact test due to small cell counts. A p-value <0.05 was considered statistically significant.

## Results

A total of 286 BPT students participated in the study. The majority were second-year students (34.62%), followed by first-year (26.92%), third-year (18.18%), fourth-year (17.13%), and interns (3.1%). Among the participants, 70.28% reported having heard about NCDs, 19.6% had not, and 10.1% were unsure. Only 16.1% of respondents had attended workshops or lectures related to NCDs.

The primary source of information was social media (51.05%), followed by faculty teaching (38.46%) and other sources such as television, newspapers, or family (10.49%). A large proportion of students (88.46%) had never participated in an NCD awareness campaign, and only 30.07% were aware of the WHO Global Action Plan (2013-2020) for NCD prevention and control. Almost all participants (97.2%) agreed that NCD content should be integrated into the physiotherapy curriculum (Table 1).

**Table 1:** Descriptive statistics and frequency of awareness items BPT, Bachelor of Physiotherapy; NCD, noncommunicable disease

Response	Category	Frequency (n)	Percentage (%)
Total number of participants (n)	BPT	286	100
Year of study	Second year	99	34.62
	First year	77	26.92
	Third year	52	18.18
	Fourth year	49	17.13
	Intern	9	3.15
Have you heard about NCDs?	Yes	201	70.28
	No	56	19.58
	Maybe	29	10.14
Have you attended workshops or lectures on NCDs?	No	240	83.92
	Yes	46	16.08
Sources of information about NCDs	Social media	146	51.05
	Faculty	110	38.46
	Others (e.g., TV, newspaper, family, etc.)	30	10.49
Have you ever attended an NCD awareness campaign?	No	253	88.46
	Yes	33	11.54
Are you aware of the WHO Global Action Plan (2013-2020) for NCDs?	No	200	69.93
	Yes	86	30.07
Should NCD education be integrated into the existing curriculum?	Yes	278	97.2
	No	8	2.8

A high proportion of students demonstrated knowledge of NCDs and associated risk factors. About 96.85% identified NCDs as a global health problem, and 95.1% recognized their prevalence in India. Most students (81.47%) recognized that the majority of premature deaths occur in low- and middle-income countries, and 85.66% agreed that NCDs account for the highest mortality rate in India. Regarding global mortality, 63.28% correctly indicated that approximately 41 million people die annually due to NCDs.

The majority (91.96%) agreed that NCDs can be considered lifestyle diseases, and 95.1% were aware of major modifiable risk factors, including tobacco use, alcohol consumption, unhealthy diet, and physical inactivity. Furthermore, 92.66% of participants recognized the role of physical activity in preventing NCDs, and 85.31% were aware of the WHO recommendation of 150 minutes of physical activity per week for adults. A large portion of respondents (90.91%) felt confident in advising patients regarding physical activity for NCD prevention, and 86.36% reported using digital health tools such as fitness apps or step counters (Table 2).

**Table 2:** Frequency and percentage of correct responses for each knowledge item * Indicates the correct response NCD, noncommunicable disease

Knowledge item	Response	Frequency (n)	Percentage (%)
Are NCDs prevalent in India?	Yes	272*	95.1
	No	14	4.9
Do you believe that NCDs are now a global public health problem?	Yes	277*	96.85
	No	9	3.15
Where do the majority of premature deaths from NCDs occur?	Low- and middle-income countries	233*	81.47
	High-income countries	33	11.54
	Not sure	20	6.99
Is NCD largely responsible for the global disease burden?	Yes	266*	93.01
	No	20	6.99
Is NCD responsible for the highest mortality in India?	Yes	245*	85.66
	No	41	14.34
How many people die globally from NCDs annually?	41 million	181*	63.28
	11 million	36	12.59
	21 million	28	9.79
	31 million	41	14.34
Is NCD projected to cause the highest number of deaths by 2030?	Yes	245*	85.66
	No	41	14.34
Which type of diabetes is most common globally?	Type 2 diabetes	236*	82.52
	Type 1 diabetes	37	12.94
	Gestational diabetes	13	4.54
Can we consider NCDs as lifestyle diseases?	Yes	263*	91.96
	No	23	8.04
Have you heard about the risk factors for NCDs?	Yes	272*	95.1
	No	14	4.9
Which of the following are modifiable risk factors for NCDs?	Tobacco use, alcohol, unhealthy diet, and physical inactivity	219*	76.57
	Only tobacco and alcohol	38	13.29
	Only diet and exercise	21	7.34
	Don’t know	8	2.8
Does diet control help in preventing NCDs?	Yes	247*	86.36
	Maybe	24	8.39
	No	15	5.25
Which are the four behavioral risk factors causing a high disease burden in India?	Tobacco use, alcohol, unhealthy diet, and physical inactivity	231*	80.77
	Only any two of the above	35	12.24
	Don’t know	20	6.99
What is the most significant risk factor for cancer in general?	Tobacco use	223*	77.97
	Unhealthy diet	41	14.34
	Alcohol consumption	22	7.69
Does physical activity help prevent NCDs?	Yes	265*	92.66
	Maybe	17	5.94
	No	4	1.4
Insufficient physical activity is a risk factor for which diseases?	All of the above (cardiovascular disease, diabetes, obesity, etc.)	263*	91.96
	Obesity	18	6.29
	Hypertension	5	1.75
What is the WHO recommendation for physical activity among children and adolescents?	60 minutes of moderate to vigorous activity daily	266*	93.01
	30 minutes per day	14	4.9
	Not sure	6	2.09
What is the WHO-recommended level of physical activity for adults (18+ years)?	150 minutes per week	244*	85.31
	120 minutes per week	28	9.79
	160 minutes per week	14	4.9
Are you confident in advising patients on physical activity for NCD prevention?	Yes	258*	90.21
	No	28	9.79
Have you used digital health tools (fitness apps, step counters, etc.)?	Yes	247*	86.36
	No	39	13.64
Do you feel confident educating patients about modifiable lifestyle changes to prevent NCDs?	Yes	260*	90.91
	No	26	9.09

Composite scoring indicated that 80.4% of students had moderate knowledge, 18.2% had poor knowledge, and 1.4% demonstrated good knowledge. Fisher’s exact test revealed a statistically significant association between knowledge level and year of study (p = 0.002), with higher proportions of moderate and good knowledge observed among students in advanced academic years (Table 3).

**Table 3:** Association between knowledge level and year of study

Year of study	Poor, n (%)	Moderate, n (%)	Good, n (%)
	N = 52	N = 230	N = 4
First year (N = 77)	21 (27.3%)	55 (71.4%)	1 (1.3%)
Second year (N = 99)	22 (22.2%)	77 (77.8%)	0 (0%)
Third year (N = 52)	6 (11.5%)	44 (84.6%)	2 (3.8%)
Final year + intern (N = 58)	3 (5.2%)	54 (93.1%)	1 (1.7%)

## Discussion

The present study assessed physiotherapy students’ knowledge and awareness of NCDs and associated lifestyle risk factors. Overall, students demonstrated a moderate level of knowledge. While most participants correctly identified NCDs as a major global health concern and recognized key modifiable risk factors, awareness-related items showed limited variability and were therefore summarized descriptively. In contrast, knowledge scores demonstrated greater variability, allowing meaningful comparisons across academic years. Notably, gaps were identified in areas related to formal training exposure, familiarity with public health guidelines, and structured preventive programs. These findings align with growing evidence that healthcare and allied health students worldwide often exhibit partial understanding of NCD-related concepts despite being future health professionals [21,22].

The questionnaire was adapted from the study by Shirotriya and Batra, which similarly reported moderate levels of NCD-related knowledge among university students, alongside gaps in exposure to structured preventive and public health initiatives [16]. Several studies report that university and allied health students frequently present multiple NCD risk behaviors, including insufficient physical activity, unhealthy diet, and high stress levels [23-25]. Similar patterns emerged in the present study, where only a small proportion of students had attended NCD-related workshops or awareness campaigns, and social media remained the primary information source. This suggests that awareness alone may not translate into active health engagement. These findings are consistent with evidence that health behaviors among students are shaped by a combination of internal factors, such as sleep, dietary habits, and mental wellness, and external factors, including academic workload and environmental influences [25,26].

The significant association between year of study and knowledge levels highlights the positive influence of academic progression and clinical exposure. Similar observations have been reported in other studies, where senior students demonstrated greater preparedness in recognizing and addressing lifestyle-related health issues [27]. However, the low awareness of global initiatives indicates limited orientation toward public health policy and population-level prevention. This gap has also been noted in previous studies across physiotherapy and allied health curricula [22,28].

The majority of students demonstrated high confidence in advising on physical activity; however, evidence suggests that many physiotherapy students and clinicians may still lack comprehensive skills in broader risk assessment, cardiovascular screening, and lifestyle counseling beyond exercise [28,29]. This discrepancy reinforces the need for competency-based education and structured health promotion training to prepare students for contemporary physiotherapy practice. Overall, the findings underscore a clear need to strengthen the physiotherapy curriculum by integrating NCD prevention frameworks, lifestyle behavior change strategies, and community health promotion opportunities.

Limitations

This cross-sectional study relied on self-reported data collected through a self-administered questionnaire and may therefore be subject to recall bias and social desirability bias. Convenience sampling and voluntary participation limit external validity and introduce potential selection and non-response bias, as the response rate could not be determined. Although institutions from both urban and rural regions were included, the sample cannot be considered representative of all physiotherapy institutions in India.

Although the questionnaire was adapted from a previously validated instrument, formal pilot testing and internal consistency reliability analysis were not conducted prior to deployment in the Indian context. Additionally, clinical competencies and curriculum content were not objectively assessed. The sample size was calculated for prevalence estimation using a single proportion formula and was not based on power analysis for detecting statistical associations. Therefore, analyses examining associated factors, including comparisons across years of study, should be interpreted as exploratory.

## Conclusions

The present study highlights that physiotherapy students from the participating universities demonstrated a moderate level of knowledge regarding NCDs and their associated lifestyle risk factors. While awareness of the global disease burden and preventive strategies was generally good, formal exposure through curricula or structured awareness programs appeared limited. Knowledge levels tended to improve with advancing academic years. These findings suggest that targeted educational initiatives focusing on NCD prevention and health promotion could enhance student preparedness within the participating institutions. Further research is recommended to assess knowledge and awareness across a broader population of physiotherapy students to strengthen evidence and inform curriculum development.

## Appendices

Questionnaire

Study title: Awareness of Non-Communicable Diseases and Inﬂuencing Lifestyle Factors Among Physiotherapy Students: A Cross-Sectional Study

Study description: This research study, "Awareness of Non-Communicable Diseases and Inﬂuencing Lifestyle Factors Among Physiotherapy Students: A Cross-Sectional Study," aims to assess the awareness levels, knowledge gaps, and behavioral patterns related to Non-Communicable Diseases (NCDs) among physiotherapy students. NCDs, such as cardiovascular diseases, diabetes, cancer, and respiratory diseases, are a leading cause of mortality globally. Understanding physiotherapy students' knowledge about these conditions is crucial as they play a vital role in promoting preventive strategies and guiding patients toward healthier lifestyles.

Purpose of the study:

To assess the awareness of NCDs and associated risk factors among physiotherapy students.

To identify common sources of information inﬂuencing students' knowledge.

To explore students' perceptions of lifestyle habits related to NCD prevention.

To obtain respondent inputs that may inform future enhancement of NCD-related education in physiotherapy curriculum.

Participant expectations:

The survey will take approximately 10-15 minutes to complete.

All responses will remain confidential and will be used solely for research purposes.

Participation is voluntary, and participants may withdraw at any point without consequence.

Your participation will help identify potential educational gaps and improve awareness initiatives to better prepare future healthcare professionals.

Consent form:

I confirm that:

I have read the project description and understand the purpose of the study.

I understand that my participation is voluntary, and I can withdraw at any time without penalty.

I am aware that my responses will be kept confidential and used solely for research purposes.

By proceeding, I agree to participate in this survey.

1. Willing to participate in the study - I agree

Education level

2. Degree program

BPT

3. Year of study

First year

Second year

Third year

Fourth year

Intern

Awareness and education on NCDs

4. Have you heard about NCDs?

Yes

No

Maybe

5. Have you attended workshops or lectures on NCDs?

Yes

No

6. Sources of information about NCDs

Social media

Newspapers

Television

Radio

Faculty

Other

7. Have you ever attended an NCD awareness campaign?

Yes

No

8. Are you aware of the WHO Global Action Plan 2013-20 for the prevention and control of NCDs?

Yes

No

9. Should NCD education be integrated into the existing curriculum?

Yes

No

Knowledge of NCDs

10. Are NCDs prevalent in India?

Yes

No

Maybe

11. Do you believe that NCDs are now a global public health problem?

Yes

No

Maybe

12. Where do the majority of premature deaths from NCDs occur?

Low-income countries

Middle-income countries

High-income countries

Not sure

13. Is NCD largely responsible for the global disease burden?

Yes

No

14. Is NCD responsible for the highest mortality in India?

Yes

No

15. How many people die globally from NCDs annually?*

15 million

25 million

41 million

16. Is NCD projected to cause the highest number of deaths by 2030?

Yes

No

17. Which type of diabetes is most common globally?

Type 1

Type 2

Risk factors and prevention

18. Can we consider NCDs as lifestyle diseases? 

Yes

No

Maybe

19. Have you heard about the risk factors for NCDs?

Yes

No

Maybe

20. Which of the following are modifiable risk factors for NCDs?

Tobacco use

Unhealthy diet

Physical inactivity

Alcohol consumption

All of the above

21. Does diet control help in preventing NCDs?

Yes

No

Maybe

22. Which of the following are major risk factors contributing to the high burden of non-communicable diseases in India?

Alcohol consumption

Obesity

Diabetes

High BP

All of the above

23. What is the most significant risk factor for cancer in general?

Unhealthy food

Alcohol consumption  

Sunlight

Tobacco use  

24. Does physical activity help prevent NCDs? 

Yes  

No  

Maybe

25. Insufficient physical activity is a risk factor for which diseases? 

Cardiovascular diseases

Type 2 diabetes

Cancer

Obesity

All of the above

26. What is the WHO recommendation for physical activity among children and adolescents?

60 minutes of moderate to rigorous activity  

70 minutes of moderate to rigorous activity  

80 minutes of moderate to rigorous activity

27. What is the WHO-recommended level of physical activity for adults aged 18+ years?

150 minutes per week  

120 minutes per week  

160 minutes per week

Practical knowledge and readiness for action

28. Are you confident in advising patients on physical activity for NCD prevention?

Yes  

No

29. Have you used digital health tools (e.g., fitness apps, step counters) to track or improve lifestyle behaviors?

Yes  

No

30. Do you feel confident educating patients about modifiable lifestyle changes to prevent NCDs?

Yes  

No

31. What strategies do you believe physiotherapy students can adopt to better educate patients about NCD prevention?
